# Reliability and validity of the workplace harassment questionnaire for Korean finance and service workers

**DOI:** 10.1186/s40557-016-0133-0

**Published:** 2016-09-09

**Authors:** Myeongjun Lee, Hyunjung Kim, Donghee Shin, Sangyun Lee

**Affiliations:** 1 Department of Occupational & Environmental Medicine, Wonjin Green Hospital, Seoul, South Korea; 2 Korean Finance and Service Workers’ Union, Seoul, South Korea

**Keywords:** Workplace harassment, Finance workers, Questionnaire development

## Abstract

**Background:**

Harassment means systemic and repeated unethical acts. Research on workplace harassment have been conducted widely and the NAQ-R has been widely used for the researches. But this tool, however the limitations in revealing differended in sub-factors depending on the culture and in reflecting that unique characteristics of the Koren society. So, The workplace harassment questionnaire for Korean finace and service workers has been developed to assess the level of personal harassment at work. This study aims to develop a tool to assess the level of personal harassment at work and to test its validity and reliability while examining specific characteristics of workplace harassment against finance and service workers in Korea.

**Methods:**

The framework of survey was established based on literature review, focused-group interview for the Korean finance and service workers. To verify its reliability, Cronbach’s alpha coefficient was calculated; and to verify its validity, items and factors of the tool were analyzed. The correlation matrix analysis was examined to verify the tool’s convergent validity and discriminant validity. Structural validity was verified by checking statistical significance in relation to the BDI-K.

**Results:**

Cronbach’s alpha coefficient of this survey was 0.93, which indicates a quite high level of reliability. To verify the appropriateness of this survey tool, its construct validity was examined through factor analysis. As a result of the factor analysis, 3 factors were extracted, explaining 56.5 % of the total variance. The loading values and communalities of the 20 items were 0.85 to 0.48 and 0.71 to 0.46. The convergent validity and discriminant validity were analyzed and rate of item discriminant validity was 100 %. Finally, for the concurrent validity, We examined the relationship between the WHI-KFSW and pschosocial stress by examining the correlation with the BDI-K. The results of chi-square test and multiple logistic analysis indicated that the correlation with the BDI-K was satatisctically significant.

**Conclusion:**

Workplace harassment in actual workplaces were investigated based on interviews, and the statistical analysis contributed to systematizing the types of actual workplace harassment. By statistical method, we developed the questionare, 20 items of 3 categories.

## Background

“Harassment” means systematic and repeated unethical acts that make someone feel insignificant as the victim cannot defend him/herself [1] and thus is assigned an inferior status [2]. Particularly, workplace harassment may be defined as acts that are repeated and conducted systematically throughout a certain period of time such as humiliation, aggressive behavior, social exclusion, and interference with one’s duty [3]. Unjust treatment can also be classified as a type of workplace harassment defined in a broad sense. Such workplace harassment has implications not only for the targeted worker but also for work productivity and economic efficiency. Furthermore, it affects the quality of the entire workforce, and thus ultimately is a deciding factor when it comes to a nation’s labor culture [4].

Research on workplace harassment have been conducted mostly in Western Europe, Austria, New Zealand, and the U.S [5, 6]. The research of Mikkelsen and Einarsen defines victims of workplace harassment as those who have experienced two or more types of negative behaviors of workmates or supervisors every week or every day for the last 6 months [7]. According to this criterion, 6.2 % of workers in Norway, 26.9 % of workers at public agencies in Finland, and 25 % of workers in the U.S. are suffering from workplace harassment [8–10].

Usually, workplace harassment occurs more often and lasts for a longer period than mere interpersonal conflicts [11]. Workplace harassment includes various types of negative experiences such as criticism from workmates (including supervisors and subordinates) regarding work, defamation of character, excessive monitoring of work, unbearable workload, unreasonable criticism, and indignation with no reason.

Workplace harassment is an important issue because exposure to such situations is known to be an immediate cause of health problems. Okechukwu et al. analyzed the effect of workplace injustice (discrimination, harassment, humiliation, etc.) on health inequality [12], and Willness et al. reported that sexual harassment was associated with PTSD, anxiety, and depressive symptom [13]. According to one line of research, depressive symptoms sometimes leads to suicide [14]. It has also been reported that merely witnessing injustice at work can cause harm to health [15].

For this reason, the need for workplace harassment assessment and intervention has been emphasized, and tools to assess workplace harassment have also been developed. One major example is the NAQ. This tool was originally developed in the Norwegian language by Einarsen and Raknes and then translated into English. Its reliability and validity were then verified [16]. Thereafter, the English version of the NAQ-R (the Negative Acts Questionnaire – Revised) was developed through representative research conducted by Einarsen and Hoel among U.K. workers in 2001 [17], and this has been widely used for research on workplace harassment in foreign countries.

However, adopting this tool directly into this study involves several problems. First, to analyze workplace harassment, the socio-cultural background of the region needs to be taken into consideration [18], and in-depth interviews to grasp the organizational culture also need to be conducted for the tool to be applied [19]. This tool(NAQ-R), however, has limitations in revealing differences in sub-factors depending on the culture and in reflecting that unique characteristics of the society [20]. Second, workplace harassment has been recognized as personal harassment by those in higher positions at work, but in consideration of current domestic conditions, the relationship between an organization and an individual also needs to be analyzed. For example, harassment may be used to put pressure upon individuals not merely for ordinary labor management but for the sake of fulfilling organizational goals such as restructuring. In recent domestic conditions, workplace harassment between a corporate organization and an individual has become an issue especially in the business area of finance and service.

In the Korean finance and service industry, the problem of job insecurity is becoming worse due to the merger and abolition of branches and workforces, an excessively performance-based system, restructuring, etc. This is not a temporary phenomenon, but it is closely related to worldwide changes in financial industry sectors. Competition, performance-based systems, and job insecurity are rampant because of deregulation, intensified pressure due to competition, introduction of new technologies, etc. Such changes in industry conditions are a major factor that brings the issue of organizational harassment against individuals to the fore.

Accordingly, this study aims to develop a tool to assess the level of personal harassment at work and to test its validity and reliability while examining specific characteristics of workplace harassment against office and finance service workers in Korea.

## Methods

### Participants and data collection

A survey was conducted among members of ‘The Korean Finance and Service Workers’ Union’ for 4 weeks from June 3, 2015, via an online survey. For this survey, focused-group inteviews were implemented fot 2 weeks from April 27, 2015 and total 10 persons attended the meeting for 3 times. The attendants were consist of different union branches.

The personal URL of each union branch was established and distributed through personal emails or mobile applications. Each personal device (computer or mobile) could be used only once for the survey, and duplicate replies were not valid. Total responses were 3065, but 1314 responses in total were used for the analysis with responses involving missing values excluded.

### Development of the harassment assessment tool

This is a methodological study to develop a tool to analyze types of workplace harassment among office and finance service workers in Korea and to verify its reliability and validity. The general framework of the survey was established based on a literature review on workplace harassment and the consultation of experts in areas of law, social studies, etc. A questionnaire was then developed specifically for members of ‘The Korean Finance and Service Workers’ Union’, and focused-group interviews were additionally conducted to examine specific harassment experiences. Interviewees included 11 individuals working at 11 subordinate unions (5 insurance agencies, 3 lending and deposit agencies, and 3 security agencies), and the interviews were conducted in a way that allowed participants to make free open-ended responses to the previously prepared questions. Based on the results, harassment was classified into 6 categories: humiliating words and deeds, harassment related to relationships, harassment related to work, supervision and control, physical harassment, and sexual harassment. A preliminary survey with the finalized items was conducted among union executive members and ordinary union members. After removal of improper terms and revision of item composition, 20 items on workplace harassment types were selected (Appendix).

### Analysis method

The reliability and validity of the developed tool was analyzed by means of the trial version of SPSS20. To verify its reliability, Cronbach’s alpha coefficient was calculated; and to verify its validity, items and factors of the tool were analyzed. KMO and Bartlett’s test of sphericity were used to verify the appropriateness of the factor analysis, and the Varimax rotation method was utilized in the factor extraction. The correlation matrix analysis was examined to verify the tool’s convergent validity and discriminant validity.

Structural validity was verified by checking statistical significance in relation to the BDI-K, a measure of depressive symptoms.

Depressive symptoms are commonly observed in clinical areas of mental health. The criterion-related validity of the questionnaire was analyzed by means of the BDI-K(The Validation Study of Beck Depression Scale 2 in Korean Version), which is commonly used with regard to depressive symptoms. The BDI-K is self-recording type questionnaire but showed a reliable level of sensitivity [21] and widely used not only for selection and asessment of patients with depression but also for screening tests for epidemiological surveys and for common populations [22]. According to existing research findings, harassment experiences have harmful effects on the workers’ mental health [23] and such situations have led even to suicide [14]. Thus, this study also aimed to uncover the relationship between the questionnaire and psychosocial stress by examining the correlation with the BDI-K.

The deficnition of harassment victims is contracted the suggestion of Mikkelsen and Einarsen. The NAQ-R The NAQ-R defines harassment victims as those who have experienced at least one type of workplace harassment at least every week during the last 6 months [10]. However, Mikkelsen and Einarsen suggestied a stricter condition:at least two two types of workplace harassment every week or every day [7].

## Results

### General characteristics of study participants

The survey results of 1314 participants in total were analyzed. As for business types, the number of workers in insurance was 820, the largest portion. Seven hundred and twenty nine individuals, 55.5 % of the participants, were women. The average age was 34.9, and the average working period was 8.7 years. Most participants(94.9 %) were college graduates or higher. 58.8 %, the largest portion, were employed in office work/supporting tasks. 45 % were section chiefs or deputy section chiefs. 33.3 % worked for more than 50 h per week on average, and 23 % worked during holidays at least once (Table 1.)

**Table 1:** General characteristics of study population

	Number	Percent
Business types		
Stock	356	27.1
Insurance	820	62.4
Finance	138	10.5
Sex		
Male	585	44.5
Female	729	55.5
Age		
20 ~ 29	272	20.7
30 ~ 39	710	54.0
40 ~ 49	319	24.3
50~	13	1.0
Last School Graduation		
High school	67	5.1
College	859	65.4
University	260	19.8
Graduate School	128	9.7
Duty		
Management	219	16.7
Office work/Supporting task	773	58.8
Sales/Service	322	24.5
Rank		
Department manager	198	15.1
Section Chief/Deputy section Chief	591	45.0
Assistant Manager/Staff	525	40.0
Working period(years)		
0 ~ 4	431	32.8
5 ~ 9	409	31.1
10~	474	36.1
Working time per week(hours)		
40	114	8.7
41 ~ 50	763	58.1
50~	437	33.3
Working day during holidays(days)		
None	1012	77.0
1~	302	23.0
Total	1314	100.0

### Reliability of workplace harassment questionnaire for Korean office and finance service workers

Regarding the reliability of the questionnaire, Cronbach’s alpha coefficient was calculated to measure internal consistency. Cronbach’s alpha coefficient of this survey was 0.93, which indicates a quite high level of reliability (Table 2).

**Table 2:** Rotated Factor Pattern Matrix and Communality of the Questionnaire

	Factor1	Factor2	Factor3	Communality
Q12	0.78			0.59
Q04	0.71			0.57
Q15	0.69			0.59
Q11	0.69			0.54
Q14	0.66			0.46
Q01	0.66			0.52
Q13	0.65			0.47
Q07	0.64			0.59
Q03	0.64			0.61
Q16	0.62			0.49
Q09	0.55			0.64
Q10	0.48			0.55
Q08	0.41			0.52
Q02		0.80		0.71
Q20		0.76		0.64
Q17		0.57		0.51
Q06		0.52		0.49
Q19			0.85	0.73
Q18			0.71	0.61
Q05			0.48	0.46
Eigen value	8.91	1.40	1.00	
Proportion of variances (%)	44.53	7.01	5.01	
KMO = 0.95; Bartlett test of sphericity = 12,785.687 (*p* < .0001)				
Total Cronbach’s α = 0.93				

### Factor analysis of workplace harassment questionnaire for Korean office and finance service workers

The questionnaire used in this study adopts the format of the NAQ-R, but the content was prepared based on actual cases of office and finance service workers. Thus, this survey tool was used for the first time in this study. To verify the appropriateness of this survey tool, its construct validity was examined through factor analysis.

The KMO test was conducted to verify sample appropriateness, and the value was 0.95, which is higher than 0.50, indicating that the factor analysis is valid. As a result of Bartlett’s test of sphericity, the Chi-square statistic was 12785.687 (*p* < 0.001) and the correlation matrix was not the identity matrix. Thus, the factor analysis was valid. Factor extraction was conducted so that eigenvalues could be at least 1.0 and the accumulated percentage of the general variance could be at least 50 %. The factor loading values and communalities of each factor item were all 0.4 or higher. As a result of the factor analysis, 3 factors were extracted, explaining 56.5 % of the total variance. The loading values and communalities of the 20 items were 0.85 to 0.48 and 0.71 to 0.46, respectively, which were all 0.4 or higher and thus met the standards [24].

Regarding individual factors, factor 1 included 13 items in total which are about “excessive workload(Q11, Q12), unjust work-related threatening(Q04, Q14) and monitoring(Q15, Q16), injustice in work assignment(Q7, Q08 Q13), work-related defamation of character(Q01, Q03 Q10), exclusion from the work process(Q09),” etc. These items may be classified as “work-related harassment.” Factor 2 includes items about “Appearance-related statements(Q02) and monitoring(Q17), sexual humiliation(Q20), and negative rumors(Q06)” and these can be classified as “defamation of character.” Factor 3 includes items about “corporal punishment(Q19), physical threat(Q18) and exclusion from fellows(Q05),” and these were classified as “physical harassment.” (Appendix).

The explanation power of “work-related harassment (factor 1)” was 44.53 %, and that of “defamation of character (factor 2)” and “physical harassment (factor 3)” was 7.01 and 5.01 % (Table 2.)

### Convergent validity and descriminent validity

The convergent validity and discriminant validity were analyzed. When the correlation coeffcient between each item and the sub-factor was at least 0.40, convergent validity was deemed satisfactory [25]. When the correlation coefficient between each item and the sub-factor was significantly different than the correlation coefficients with items that did not belong to it, discriminant validity was deemed satisfactory. As shown in Table 3, correlation coefficients between each of the 20 items and factors that each of them belonged to were between 0.41 and 0.85, all of which exceeded 0.40. Thus the success rate of item discriminant validity was 100 %. Likewise, the correlation coefficient between other sub-factors that each item did not belong to was between −0.01 and 0.48, and no value exceeded the correlation coefficient with factors that each item belonged to (Table 3.)

**Table 3:** Multi-trait/multi-item matrix of the questionnaire (Correlation matrix corrected for overlap)

		Factor1	Factor2	Factor3
Factor1	Q01	0.66	0.34	0.20
	Q03	0.64	0.36	0.17
	Q04	0.71	0.15	0.27
	Q07	0.64	0.29	0.19
	Q08	0.41	0.40	0.37
	Q09	0.55	0.39	0.26
	Q10	0.48	0.14	0.47
	Q11	0.69	0.33	0.03
	Q12	0.78	0.05	0.05
	Q13	0.65	0.26	0.09
	Q14	0.66	0.44	0.12
	Q15	0.69	0.18	0.19
	Q16	0.62	0.22	0.30
Factor2	Q02	0.23	0.80	0.14
	Q06	0.44	0.52	0.15
	Q17	0.38	0.57	0.20
	Q20	0.14	0.76	0.21
Factor3	Q05	0.27	0.40	0.48
	Q18	0.26	0.22	0.71
	Q19	−0.01	0.11	0.85

### Criterion-related(concurrent) validity

This study also aimed to uncover the relationship between the questionnaire and psychosocial stress by examining the correlation with the BDI-K.

The questionnaire included 20 questions in total, each of which was differentiated depending on the frequency of harassment experiences during the last year (Appendix). Each answer to these questions was given points from 1 to 5, and the BDI-K was divided into 4 steps(minimal, mild, moderate, severe) to examine the statistical correlation through the Chi-square test. As a result, 19 items out of 20 (the 19th item being the exception, *p* = 0.3320) showed statistically significant correlations with depressive symptoms, whose level increased in proportion to the frequency of harassment experiences (*p* < 0.0001).

Mikkelsen and Einarsen defines harassment victims as those who have experienced at least two types of workplace harassment every week or every day [7]. This condition is widely used, the questionnaire adopts it in examining statistical significance in relation to the BDI-K. As a result, it was found that about 15 % of the respondents were exposed to serious workplace harassment, and that this harassment was significantly related to the severity of depressive symptoms (*p* < 0.001). Answers to the 20 items were scored as none (1 point), less than once a month (2 points), once a month (3 points), once a week (4 points), and almost every day (5 points), and the total score was divided into quartile values in order to examine its relationship with the BDI-K. In this case, the 25, 50, and 75 percentile scores were 20, 23, and 30, respectively, which indicates that the correlation with the BDI-K was statistically significant (*p* < 0.001) (Table 4).

**Table 4:** The association of distribution of Strength of harassment group by the questionnaire and depressive symptom group

		BDI-K			
	N	Minimal	Mild	Moderate	Severe
The questionnaire distribution (*p* < 0.001)					
0 ~ 25 %	350 (26.64 %)	260 (74.29 %)	44 (12.57 %)	29 (8.29 %)	17 (4.86 %)
25 ~ 50 %	321 (24.42 %)	203 (63.24 %)	62 (19.31 %)	45 (14.02 %)	11 (3.43 %)
50 ~ 75 %	348 (26.84 %)	146 (41.95 %)	80 (22.99 %)	95 (27.3 %)	27 (7.76 %)
75 ~ 100 %	295 (22.45 %)	80 (27.12 %)	63 (21.36 %)	93 (31.53 %)	59 (20 %)
Total	1314 (100.00 %)	689	249	262	114
Strength of Harassment (*p* < 0.001)					
Weak	1116 (84.94 %)	642 (57.53 %)	208 (18.64 %)	202 (18.1 %)	64 (5.73 %)
Strong	198 (15.06 %)	47 (23.74 %)	41 (20.71 %)	60 (30.30 %)	50 (25.25 %)
Total	1314 (100.00 %)	689	249	262	114

After some corrections to items regarding sex, age, married/single, duties, working hours, and working on weekends, multiple logistic analysis was conducted. Despite corrections to the items, those with severe harassment experience showed 4 times more depressive symptoms than those without when the NAQ-R criteria were applied (OR = 4.01, CI = 3.00 ~ 5.36). In the quartile classification as well, the top 25 % showed 7 times more depressive symptoms than the bottom 25 %(OR = 7.20, CI = 5.19 ~ 10.00) (Table 5.)

**Table 5:** The result of multiple logistic analysis of the association the questionnaire and the depressive symptoms

	OR	95 % CI	
The questionnaire distribution			
0 ~ 25 %	1.00		
25 ~ 50 %	1.58	1.14	2.19
50 ~ 75 %	3.73	2.73	5.09
75 ~ 100 %	7.20	5.19	10.00
Strength of harassment			
Weak	1.00		
Strong	4.01	3.00	5.36

This result was adjusted by sex, age, marriage, duties, working hours and working on weekends

## Discussion

When it comes to research on the issue of workplace harassment, it is vital to consider both sociocultural backgrounds and peculiarities of assigned duties. Although the NAQ-R is certainly a verified harassment assessment tool that has long been used, it has limitations in reflecting the unique situation in Korea. Additionally, survey tools that have been developed in other lines of research are also limited to certain occupational groups such as nurses, and it may be inappropriate to apply them to office and finance service workers in Korea (members of the Korean Finance and Service Workers’ Union’). Since there was no specialized tool that can be used, an assessment tool specialized for office and finance service workers in Korea was developed in this study.

NAQ-R survey the frequency of harassment and bullying without the definition of workplace harassment and being victimized [7, 26]. That is toal 20 items, which is composed 3 sub categories (personal related bullying(12 items), intimidation related bullying(5 items), work-related bullying(5 items).

However, this assessment tool had 6 subcategories in the initial stage of design: “humiliating words and deeds,” “relationship-related harassment,” “work-related harassment,” “supervision and control,” “physical harassment,” and “sexual harassment.” This tool is more specific questionnaire for Korean finance and service workers, because the each categories are based on the in-depth interview form their experiences.

These 6 subgroups were reduced to 3 after factor analysis: “work-related harassment, “defamation of character,” and “physical harassment.” Twenty items satisfied the standards for loading and communality, explaining 56.6 % of the total variance. Cronbach’s alpha coefficient was 0.93, which indicates a high level of internal consistency.

The correlation between workplace harassment and individual depressive symptoms was examined by means of the BDI-K. As a result, it was shown that those who were exposed to two or more types of workplace harassment every week or every day showed an odds ratio as high as 4.02(CI = 3.01 ~ 5.37), which was higher than those who were not. In addition, scores from 1 to 5 points were given depending on the number of experiences, and the total score was converted to quartile values to examine the relationship with depressive symptoms: In the case of the 75th percentile or higher, the odds ratio was as high as 7.16 (CI = 5.16 ~ 9.94).

The questionnaire included some items that might lower the content consistency: 2 items of “physical harassment” (No. 18 and 19), 1 item of “sexual harassment” (No. 20), and 1 item of “work-related excessive demand” (No. 8). These items also represent types of workplace harassment. Although these too are serious problems at work, their frequency was not relatively high, and these were problems of personal character rather than duties at work. Thus, they might have lowered the consistency of the survey.

However, the rest of the items properly reflected kinds of workplace harassment that office and finance service workers in Korea might suffer during the work process, and these items take into consideration not only personal harassment but also harassment problems related to organizational cultures such as excessive workload upon changes in working conditions, understaffing, and excessive monitoring.

The relationship with the BDI-K was also clearly shown, verifying the statistical relationship between the level of exposure to workplace harassment and depressive symptoms. Among the relationships between the 3 factors and the BDI-K, “work-related harassment,” which was classified as factor 1, showed an especially significant positive correlation with depressive symptoms.

This survey tool, however, has several limitations: the survey was not conducted with other related surveys that could verify its external validity as part of research on the actual conditions of workplace harassment; even though the 6 categories in the questionnaire used different numbers of items along with the interviews, no corresponding weight was applied; and the participant ratio was not adjusted depending on the region. Additionally, the causal relationship between workplace harassment and depressive symptoms was not clarified because this was a cross-sectional study.

Nonetheless, types of workplace harassment in actual workplaces were investigated based on interviews, and the statistical analysis contributed to systematizing the types of actual workplace harassment. In addition, the survey was conducted not in the context of one certain workplace in Korea but over a more general set of office and finance service workers. With all these achievements, this study is of great significance. Furtheremore, there was a statistical relationship between harassment experience and depressive symptoms, and the existing study finding that experience of violence at work is related to depressive symptom was reconfirmed [23].

## Conclusion

This study develop a tool to assess the workplace harassment, consised with 20 items. By the statistical method, the validity and reliability obtatined and the correlation with depressive symptom was significant. Based on the above study results, additional research needs to be conducted to determine appropriate cut-off points for proper selection of workers who need intervention. In addition, future research also needs to expand its scope to cover general office workers.

## Appendix

**Table 6:** Workplace harassment questionnaire for Korean office and finance service workers

How often did you experience the following situations at your workplace over the past year?	Never	Less than once a month	About once a month	About once a week	Almost everyday
(Q01) I was humiliated or yelled at in front of others.	①	②	③	④	⑤
(Q02) I was insulted with demeaning expressions regarding my appearance or behavioral characteristics.	①	②	③	④	⑤
(Q03) I was offended with swearing or sarcastic verbal abuse.	①	②	③	④	⑤
(Q04) I was threatened that I would be discharged or have a disadvantage for promotion if I did not obey orders.	①	②	③	④	⑤
(Q05) I was not invited for or treated as if I did not exist at meetings or social gatherings with colleagues.	①	②	③	④	⑤
(Q06) Someone talked behind my back or spread negative rumors about me.	①	②	③	④	⑤
(Q07) I was ordered to work on tasks that were not related to my qualification, skills, or experience.	①	②	③	④	⑤
(Q08) My work was replaced by menial tasks or I was rarely assigned jobs.	①	②	③	④	⑤
(Q09) I was not informed about important messages related to work or I was not provided with necessary equipment.	①	②	③	④	⑤
(Q10) I was forced to apologize or write apology letters for mistakes in my work or other happenings beyond my control.	①	②	③	④	⑤
(Q11) Stressful tasks or others’ work were assigned to me.	①	②	③	④	⑤
(Q12) I was assigned with work that could not be completed or had close deadlines (including work assigned just before leaving work).	①	②	③	④	⑤
(Q13) I was forced to participate in meetings, events, and trainings regardless of my intention.	①	②	③	④	⑤
(Q14) I was picked on or got involved in arguments for small things.	①	②	③	④	⑤
(Q15) My work was monitored excessively.	①	②	③	④	⑤
(Q16) I was asked or it was checked in detail what I did outside of my working hours (including checking the CCTV and in-house bulletin board, and tailing).	①	②	③	④	⑤
(Q17) I was monitored and controlled for my appearance and the clothes that I wear.	①	②	③	④	⑤
(Q18) I experienced physical violence or threats (including threats imposed by throwing things).	①	②	③	④	⑤
(Q19) I was physically punished (including squat walk).	①	②	③	④	⑤
(Q20) I was sexually humiliated with words or actions.	①	②	③	④	⑤
